# Cleanliness in context: reconciling hygiene with a modern microbial perspective

**DOI:** 10.1186/s40168-017-0294-2

**Published:** 2017-07-14

**Authors:** Roo Vandegrift, Ashley C. Bateman, Kyla N. Siemens, May Nguyen, Hannah E. Wilson, Jessica L. Green, Kevin G. Van Den Wymelenberg, Roxana J. Hickey

**Affiliations:** 1 Biology and the Built Environment Center, University of Oregon, Eugene, OR USA; 2 Institute of Ecology and Evolution, Department of Biological Sciences, University of Oregon, Eugene, OR USA; 3 Energy Studies in Buildings Laboratory, Department of Architecture, University of Oregon, Eugene, OR USA

**Keywords:** Hygiene, Microbiota, Microbiome, Skin, Microbial ecology, Hand hygiene

## Abstract

The concept of hygiene is rooted in the relationship between cleanliness and the maintenance of good health. Since the widespread acceptance of the germ theory of disease, hygiene has become increasingly conflated with sterilization. In reviewing studies across the hygiene literature (most often hand hygiene), we found that nearly all studies of hand hygiene utilize bulk reduction in bacterial load as a proxy for reduced transmission of pathogenic organisms. This treatment of hygiene may be insufficient in light of recent microbial ecology research, which has demonstrated that humans have intimate and evolutionarily significant relationships with a diverse assemblage of microorganisms (our *microbiota*). The human skin is home to a diverse and specific community of microorganisms, which include members that exist across the ecological spectrum from pathogen through commensal to mutualist. Most evidence suggests that the skin microbiota is likely of direct benefit to the host and only rarely exhibits pathogenicity. This complex ecological context suggests that the conception of hygiene as a unilateral reduction or removal of microbes has outlived its usefulness. As such, we suggest the explicit definition of hygiene as “those actions and practices that reduce the spread or transmission of pathogenic microorganisms, and thus reduce the incidence of disease.”

## Background

This review focuses on the concept of hygiene as it relates to the human-associated microbiota, with the aim of coming to a clear, workable definition of hygiene that is congruent with our emerging understanding of the intimate, multifaceted, and symbiotic relationships that humans have with microorganisms. After conducting a thorough review of peer-reviewed literature on hand hygiene, the skin microbiota, handwashing, and hand drying (compiling over 200 papers), we systematically examined both clinical and commonplace definitions of hygiene and re-evaluated the concept in the context of a modern understanding of human-associated microbial ecology. By doing this, we bridged a gap between the clinical skin microbiology literature and the emerging human-associated microbial ecology literature.

Given the intimate interactions between humans and our microbiota, it is becoming apparent that maintenance and promotion of healthy human-associated microbial communities is necessary for good health. As such, we argue that the concept of hygiene as akin to sterilization no longer serves a useful role in scientific or medical discourse. It is more useful to explicitly define hygiene in terms of health outcomes and focus on the use of quantitative, modern molecular biology tools to elucidate the complex ecological interactions that relate hygienic practice to the spread of disease. Pursuant to that goal, we have explicitly defined hygiene as “those actions and practices that reduce the spread or transmission of pathogenic microorganisms, and thus reduce the incidence of disease.”

## The current definition of hygiene

The word *hygiene* originates with Hygieia, the Greek goddess of health. The Oxford English Dictionary (OED) defines it as: “That department of knowledge or practice which relates to the maintenance of health; a system of principles or rules for preserving or promoting health; sanitary science” [1]. The OED also gives us some context of the use of the word in English, noting that its origins lay with the first part of the definition (early use of the word relates entirely to the practice of medicine), while more modern usage tends to refer specifically to the practice of cleanliness where it relates to maintaining good health. In practice, however, hygiene is rarely explicitly defined. The term most often refers to *hand hygiene*, which the World Health Organization defines as “a general term referring to any action of hand cleansing” [2]. Hygiene may also refer to *environmental hygiene*, which can mean either the cleaning of surfaces within a person’s (most commonly a patient’s) environment [3] or, more broadly, infrastructural changes that alter the environment in a way perceived as beneficial to human health (such as the installation of water and sewage treatment facilities) [4]. In this review, we focus primarily on hand hygiene, since this aspect of hygiene is most commonly used in the modern scientific literature.

Despite early recognition of the importance of hand hygiene for controlling the spread of disease (Table 1) [5–7], little attention was paid to the particulars for most of the twentieth century. Though the CDC gradually increased the regulation of hand hygiene practices [8–10], particularly in healthcare settings, it was not until 2009 that an international standard for hand hygiene practices was established by the World Health Organization (WHO) in the comprehensive *Guidelines on Hand Hygiene* [2].

**Table 1:** History and hand hygiene

Interest in hand hygiene dates to the middle of the nineteenth century. Oliver Wendell Holmes, in Boston, and Ignaz Philipp Semmelweis, in Vienna, both noticed the contagious nature of puerperal fever, which affects women shortly after childbirth [5, 129]. Publishing their findings nearly concurrently, but on different continents, they both argued that physicians with unwashed hands spread the disease to birthing women. Semmelweis’s work went one step further; he made the connection that medical students often went straight from the autopsy theater to the birthing room and concluded that they must be transmitting “cadaverous particles” from the corpses to the patients. To combat this spread, he instituted a policy of scrubbing the hands in chloride of lime (calcium hypochlorite) for anyone moving between the autopsy theater and the maternity wards; mortality rates were quickly reduced [6].
Both physicians were ridiculed for their beliefs at the time, but they laid the foundations for thought about hygiene and the spread of infection in the medical establishment. Semmelweis turned to alcohol as his calls for reform were consistently ignored and refused, and was eventually tricked into entering an insane asylum. When he tried to escape, he was severely beaten, and died 2 weeks later from a gangrenous wound, probably a result of the beating [5]. Around this time, in France, Louis Pasteur was working on germ theory and fermentation, formally publishing the pasteurization method in 1865 (the year of Semmelweis’s death), followed by the initial publication on germ theory in silkworms in 1870, just 9 years after Semmelweis’s research on puerperal fever [130]. Pasteur was also working on puerperal fever; in 1880, he published microbiological observation and recommendations concerning the disease [7], which were more readily accepted by the medical establishment than Semmelweis’s recommendations.

The WHO explicitly defines hand hygiene as “any action of hand cleansing” and then goes on to delineate many specific “hand hygiene practices,” which include everything from soap and water handwashing to surgical hand antisepsis. It is noteworthy that most regulations and recommendations concerning hand hygiene focus on the aspect of hygiene as the act of cleaning, concentrating on the reduction in bulk microbial load, rather than the reduction in transmission of infection.

### Current research on hand hygiene

The focus on bulk reduction in microbial load is apparent in the majority of studies of hygiene—even those conducted by clinical microbiologists [11–14]. The concepts of hygiene and sterilization are often conflated, which is perhaps unsurprising given the history of hospital sanitation practices, which seek to remove all microbes from the environment [8]. There is a logical link between bulk reduction in microbial load and reduction in pathogen spread; however, relatively few studies go beyond cleaning and link hygiene directly to health outcomes, and many of these are specifically concerned with nosocomial infection [12].

Hand hygiene research has focused largely on hospital settings and the spread of nosocomial infection (reviewed in [2, 12]), in part due to the history of the field but moreover because of recognition of the increased risk of infection in places where potentially contagious and immunocompromised people are gathered. Where work on hand hygiene has taken place outside of hospital settings, it has focused on other areas with high risk of pathogen transmission, such as childcare facilities [15–17] or food handling situations [18], or has been undertaken in combination with efforts to improve community environmental hygiene, such as improved sanitation infrastructure in developing countries (reviewed in [19]).

Aiello and Larson found only 53 studies published between 1980 and 2001, out of thousands of studies matching their search criteria, that explicitly linked hygiene to health outcomes outside of healthcare settings [19]. Studies linking hygiene intervention to health demonstrate the effectiveness of handwashing at reducing the risk of diarrhetic disease [20, 21] and upper respiratory infection [21, 22]. Reduction in the rates of handwashing in response to fears of lead contamination have been suggested as a factor contributing to a recent *Shigella* outbreak in Flint, Michigan [23].

The lack of a clear and consistent definition of hygiene has led to confusion in the scientific literature. One example of this confusion is apparent across the hygiene literature related to *hand drying*. Though previously ignored [24], hand drying is a crucial aspect of hand hygiene because of the significant role that residual moisture plays in the transfer of microbes between surfaces [25–28]. The different methods of hand drying have varying hygienic advantages and concerns. For example, drying with paper towels is the method recommended for healthcare workers by both the Centers for Disease Control and Prevention [29] and the WHO [2], due in large part to bulk bacterial count data indicating that paper towels are effective at removing transient surface bacteria [24, 30–35]. Nevertheless, it is possible that waste paper towels may serve as a bacterial reservoir that may facilitate the spread of disease [36, 37]. Newer alternatives to paper towels such as jet air dryers (e.g., the Dyson Airblade™) are marketed as designed with a high-efficiency particulate air (HEPA) filter built into the airflow system, which reduces the risk of redistribution of airborne microbes to the hands [13]. However, there is concern about the propensity of such rapid air movement to aerosolize microbes from the users’ hands or the surrounding environment [14, 34, 37–39].

Much of the existing work on hand drying has examined the hygienic efficacy of various methods. What is meant by “hygiene” in any given study, however, is often left unstated and is generally inconsistent between studies from different research groups [40] but usually is measured by change in microbial load [13, 34], dispersal of microbes from the hands or some proxy thereof [38, 39], and/or effectiveness of drying [13, 36, 37]. Utilizing a definition of hygiene that explicitly relies on reduction in disease spread and takes into account microbial community dynamics would allow future experiments to adequately address the possible hygienic concerns of paper towel bacterial reservoirs or microbes aerosolized by dryers.

## The human microbiota and hygiene in an ecological context

**Fig. 1:**
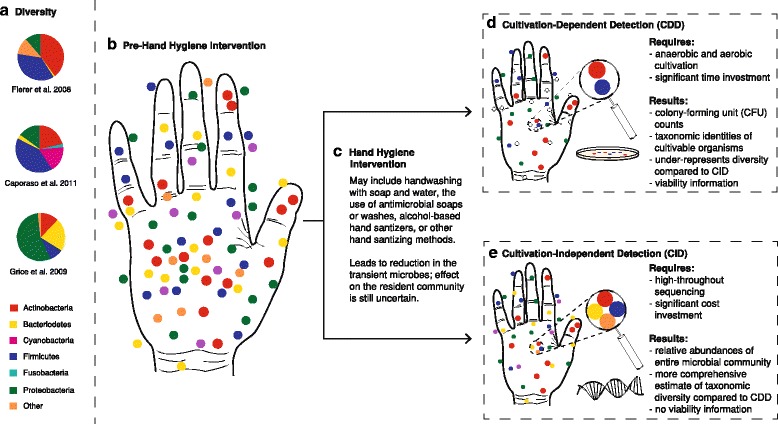
**a** Diversity of the hand microbiome is variable, with different studies finding different dominant groups (though Proteobacteria and Actinobacteria are common members of the hand microbiome across studies): data presented from [52, 54, 57]. Interactions between taxa may modulate their ecological roles, and community variation across a range of ecological traits may be altered by changes in community membership or structure [124]. **b** Pre-hand hygiene intervention. Both resident and transient microbes are abundant on the hands (in this conceptualization, *colored dots* represent individual microbes: many colors are used to show the diversity of microbes present on the hands). **c** Hand hygiene intervention, such as washing with soap and water, reduces the total number of microbes (microbial load) present on the hands. **d** Cultivation-dependent detection (*CDD*) is commonly used to study aspects of hand hygiene; cultivation yields data showing changes in the numbers of colony-forming units (counts), though many microbes are not detectable using this methodology (represented as *non-colored*, *open circles*). Some studies identify colonies using morphological or molecular methods, yielding limited taxonomic information. **e** Cultivation-independent detection, including high-throughput DNA sequencing, is commonly used to study the microbial ecology of the skin. Using these methods, it is possible to quantify alterations in relative abundance of bacterial populations with treatment (such as handwashing), to obtain deep, comprehensive taxonomic diversity estimates. Depending on technique, it may also be possible to also obtain information on functional metabolic pathways (using metagenomics and metatranscriptomics)

Most of the existing literature and the prevailing understanding of hygiene in general are based on cultivation-dependent studies (Fig. 1d), which entail the growth and enumeration of bacteria in the laboratory, and often is based solely on counts of colony-forming units (CFUs) with limited or no taxonomic information. These techniques fail to account for the high abundance and ubiquity of non-harmful—and potentially helpful—bacteria on the human skin [41, 42]. Modern cultivation-*independent* techniques (Fig. 1e), including high-throughput DNA sequencing technology, have facilitated a deeper exploration of microbial diversity and expanded our understanding of the trillions of bacteria, fungi, and viruses living on the healthy human body, collectively known as the *microbiota*, and their role in maintaining health [43–46]. Despite the skin being the largest organ in terms of surface area (1.8 m^2^), the skin microbiota is far less studied than other parts of the human microbiota (e.g., gut microbiota), and the non-bacterial inhabitants of the skin are even less well characterized, in large part due to methodological issues, perceived rarity, and asymptomatic nature [47–49]. Studies that focus on hygiene should take this diversity into account and recognize that not all microbes are harmful and that there is a *continuum* between pathogenic and mutualistic microbes.

### Skin habitat and microbial diversity

The human skin may be open to colonization from the environment, but it is thought to be a strong selective filter, largely unsuitable for most microbes to permanently reside [50]. The three major skin habitats (sebaceous, dry, moist), and the gradations of environmental conditions within and between them, largely determine the bacterial community living at a particular skin site [51, 52]. Skin bacterial communities, therefore, appear to have generally predictable biogeographic patterns. The normal/healthy skin microbiota is composed of a limited number of types of bacterial species (mainly Gram-positive species) [50, 52–55]. Dry regions, such as the forearm and palm, are often the richest in bacterial diversity, are generally less restricted in membership, and are more susceptible to temporal variability, while the sebaceous sites are generally poorer in bacterial diversity and dominated by *Propionibacterium acnes*, presumably due to high sebaceous gland activity that may result in more exclusivity [52, 53, 55, 56].

While there is some similarity in microbiota of similar body habitats and across individuals, it is abundantly clear that not all skin communities are alike [52, 53, 55, 57, 58]. There is some evidence of persistent community members at specific skin sites, with a few taxa being detected from the same site for up to a year [57, 59]. Nevertheless, it is difficult to define a *core microbiota* for a given anatomic site on the skin [60]. One review listed the most “common” human skin bacterial residents as: *Staphylococcus*, *Corynebacterium*, *Propionibacterium*, *Micrococcus*, *Streptococcus*, *Brevibacterium*, *Acinetobacterium*, and *Pseudomonas* [61]. Many of these (e.g., *Staphylococcus aureus* and *Staphylococcus epidermidis*) also have the potential to become multidrug-resistant pathogens, emphasizing the insidious nature of classifying microorganisms as one ecological mode (e.g., commensal vs. pathogenic) and the need for a conceptual framework taking into account the existing ecological continua.

### The mutualist–pathogen continuum

Much of the scientific literature related to the human skin microbiota utilizes two different sets of vocabulary: *resident* and *transient* microbes (often used in the clinical literature; e.g., [59]) vs. *commensal* and *pathogenic* microbes (often used in the ecological literature; e.g., [62]). However, these terms are rarely defined and are frequently conflated; residents are often assumed to be commensal, and transients are often assumed to be potential pathogens.

Historically, *resident* microbes were thought of as those that were stable on the human skin and were difficult to remove, whereas *transient* microbes were thought to be acquired by contact and could be easily removed from the skin [63]. This notion of resident/transient microbes has continued for decades and has morphed into the assumption that resident microbes are those that commonly reside on skin whereas transient microbes are viewed as contaminants [64].

*Commensal* microbes on the human skin are regarded as those that are not typically associated with disease [61]. However, the ecological definition of commensalism refers to the condition where only one organism receives benefit and the other organism suffers no harm [61]. The use of the term “commensal” to describe non-harmful microbes on the skin is suggestive that only the microbe is receiving benefit from living on the skin’s surface and no benefit is provided to the human host. However, this definition is misleading because there is growing evidence that microbes once thought to be commensal may actually be involved in host defense, which would suggest a *mutualistic*, rather than a commensalistic, relationship [61]. In comparison, a *pathogenic* microbe on the skin is one that causes harm to the host. There are, however, many microbes that are associated with disease which exist as normal members of the skin microbiota in healthy individuals. Taking a lesson from ecology, it is necessary to consider the host, the microbe, and the environmental context to assess the relationship of any particular microbe to a disease state [65]. When the ecological relationship between host and microbe is unclear (that is, when it is impossible to say if a given microbe is acting as a mutualist, a commensal, or a pathogen), we prefer the term *symbiont* (literally, “together living”), which does not imply an ecological mode.

Both of these dichotomies represent *continua*, which are related but orthogonal to each other. The idea of a mutualist–pathogen continuum has been successfully applied in the plant microbial ecology literature for decades [66, 67]. This continuum represents a position in niche space, which can change through alterations to microbial or host genetics, environmental conditions, and community context [53, 61, 68, 69]. The resident–transient continuum represents a temporal dimension and is defined by the length of time that a given microbe is associated with its host—though we must consider the effect of the limits of detection with current techniques [57]. It is important to recognize that resident does not necessarily equate to commensal nor does transient always mean pathogenic.

It is possible for the human skin to have mutualistic, commensal, and pathogenic microbes as part of its resident “core” microbiota; a single microbial species may be all of these things. For example, the bacterium *S. epidermidis* is commonly found on the human skin and is generally regarded as commensal [61], although it can occasionally act as an opportunistic pathogen [53, 68] or a protective mutualist [70]. Recent evidence suggests that *S. aureus*, which has been typically thought of as a pathogenic microbe, is commonly present on the healthy skin, specifically in the nasal area [71]. Following this logic, it is likely that many of the transient microbes that people are exposed to in the environment are not only non-pathogenic, but in fact could be beneficial to the skin microbiota.

### Microbial ecology of the skin

**Fig. 2:**
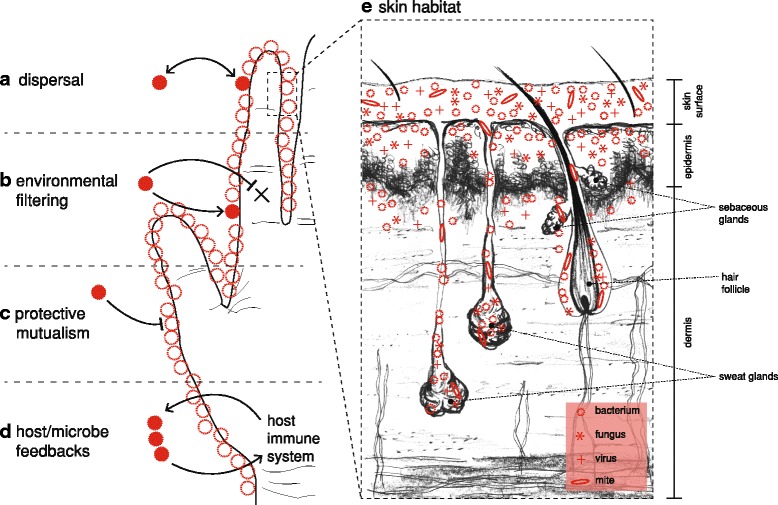
Conceptual illustration of important ecological factors impacted by hygienic practice. **a** Dispersal is the movement of organisms across space; a patch of habitat is continuously sampling the pool of available colonists, which vary across a variety of traits (dispersal efficiency, rate of establishment, ex host survivability, etc.) [125]. **b** Environmental filtering works on the traits of dispersed colonists—microbes that can survive in a given set of environmental conditions are filtered from the pool of potential colonists [125]: the resources and conditions found there permit the survival/growth of some organisms but not others. **c** Protective mutualisms function through the occupation of niche space; harmful microorganisms are excluded from colonization via saturation of available habitat by benign, non-harmful microbes [126]. **d** Host/microbe feedbacks occur via the microbiota’s ability to activate host immune response and the host immune system’s ability to modulate the skin microbiota [106, 127, 128]. Such feedbacks between host immune response and the skin microbiota are thought to be important to the maintenance of a healthy microbiota and the exclusion of invasive pathogenic microbes [87]. All of these ecological factors are affected by features of the **e** skin habitat, which includes appendages such as hair follicles, sebaceous glands, and sweat glands; microbes (bacteria, fungi, virus particles, and skin mites) reside not only on the surface but deeply within glands and the roots of hairs, as well as within the squamae of the epidermis (illustration redrawn from [53])

There is an emerging appreciation of the microbial ecology of the skin. *Community ecology* seeks to understand what factors determine the presence, abundance, and diversity of species in a community [72]. Island biogeography theory [73], in particular, allows us to conceptualize each person as an island: a patch of habitat that must emerge and assemble its communities by the fundamental processes of community ecology. The interactions between skin microbial communities and the host make understanding the ecological factors contributing to microbial communities particularly important. Multiple ecological factors interact to determine the species composition in a given ecological community; *dispersal* (Fig. 2a) and *environmental filtering* (Fig. 2b) are the two factors most relevant to the discussion of hygiene.

Dispersal (Fig. 2a) of commensal or mutualistic organisms may be particularly relevant to human health. Studies that have examined the transmission of human-associated microorganisms have almost exclusively focused on pathogenic microbes in healthcare settings [72]. The transmission of other (i.e., non-pathogenic) members of the skin microbiota is poorly understood, including the roles of a number of factors (e.g., diversity, interspecies interactions, host factors, environmental factors) on the ease of microbial transfer and subsequent colonization. Transmission via direct contact with other individuals, or indirectly with fomites or water droplets, introduces transient microbes that could alter the ecological dynamics of the skin microbiota [72].

All persons are dispersers of their microbiota, though dispersal rates vary within and among people. Organisms living on the skin have the potential to be dispersed as a result of normal desquamation (i.e., shedding or peeling of the outermost layer of the skin) [74]. Individuals emit a personalized microbial cloud that likely impacts both cohabitants and the microbiota of the built (indoor) environment itself [75]. While research on whether resident microorganisms can be transferred among individuals is nascent, it is hypothesized that delivery method at birth (vaginal vs. Cesarean section) affects initial skin microbial communities of infants [76] (but see [77]).

Environmental filtering (Fig. 2b) of dispersed microbes functions primarily through differences in skin habitat (e.g., dry vs. sebaceous sites). Interactions between microbial populations may be part of the “filtering” of the environment; thus, *priority effects* [78, 79] and factors related to the established microbiota can be considered part of the environment to newly dispersed microbes. There is evidence that host factors vary in the ability to promote bacterial colonization and that this varies by skin site [55]. The role of *host/microbe feedbacks* (Fig. 2d) in determining environmental selective pressures may also influence the outcomes of potential dispersal events. There is some evidence that microbial communities may be transferred between people or their environments [80, 81].

*Invasion ecology* focuses on perturbations of established communities and attempts to understand the factors that allow invasion by exogenous species [82, 83]. Applied to the skin microbiota, *disturbance* (e.g., hand hygiene practices) may be a major factor in alterations of the skin microbiota through invasion; *protective mutualisms* (Fig. 2c) may be disrupted or eliminated, allowing invaders to colonize. While the microbial ecosystem of the skin is relatively stable in the face of continual desquamation of the skin surface and frequent perturbations [58], disruptions by antibiotics, handwashing, or cosmetic application may alter the microbial community, enabling invasion of pathogenic microbes or a shift in dominance leading to dysbiosis [72]. Skin disturbance can predispose the host to a number of cutaneous infections and inflammatory conditions [61]. Despite the multitude of studies emphasizing the benefits of personal hygiene on reducing disease transmission by removing or reducing transient microorganisms, the effects of personal hygiene (e.g., handwashing) on the *resident* microbiota are not well studied [46]. Different behavioral habits (e.g., frequency and duration of washes, product used) likely account for at least some of the variation in microbial community structure and membership observed in human studies [72]. The frequency and magnitude of such disturbances likely facilitate the invasion of potentially undesirable, pathogenic species [84, 85].

A conceptual framework for understanding the interactions between the skin microbiota, the human host and environment, and the impact on human health must take into account all of these ecological factors [72, 84]. Significant and potentially harmful alterations of the skin microbial community structure may occur as a result of several factors: dispersal of non-resident microbes to the host microbiota, disturbance regimes (e.g., handwashing practices), local and regional environmental factors (i.e., environmental selective filters on source and sink populations, such as host skin condition and indoor settings), and the genetics and demographic characteristics of the host, which also provide selective filtering [72].

### Skin microbiota and host immunity

The resident microbiota has evolved in conjunction with the human host and is thought to be important to the maintenance of healthy ‘normal’ skin function. Generally, the resident microbiota have a positive effect on human health through protective mutualism (Fig. 2c); it is only when the host becomes compromised that the resident microbiota displays pathogenic potential [86, 87]. As the skin is our body’s interface with the outside world, it must act to both prevent colonization by pathogens and tolerate or encourage the presence of potentially protective bacteria. The skin is a complex immunological organ with both innate and adaptive immune cells, including multiple dendritic and T cell subsets: antimicrobial peptides, proinflammatory cytokines, and chemokines that are secreted by keratinocytes to support an immune response [88–93]. While pathways related to infection response are relatively well understood [56, 61, 87], the mechanisms by which commensal or transient bacteria are tolerated by the cutaneous immune system are only beginning to be understood [93].

Host–microbe feedbacks (Fig. 2d), modulated through the host immune system, have been recently demonstrated and are likely to play critical roles in maintaining healthy host/microbiota relationships. For example, *S. epidermidis* has been shown to produce antimicrobial peptides and may modulate the host immune response [94]; *S. epidermidis* and *Corynebacterium* spp. are capable of reversing or preventing the successful colonization and proliferation of *S. aureus* in the human nares [47], such that removal of *S. epidermidis* may be harmful to the host through increased colonization of opportunistic pathogens [61]. Additionally, some studies have shown that microbial exposure—particularly early in life—may have long-term implications for health and immunity (known as the hygiene hypothesis [95, 96], but see [97]). Skin dysbiosis has been linked to many skin disorders, including acne vulgaris, psoriasis, and atopic dermatitis [98–106]. Investigation of the potential of microbiota transplants and probiotic skin treatments for these diseases are underway [53]. Thus, the skin microbiota is likely of direct benefit to the host and only rarely exhibits pathogenicity.

### Microbial ecology applied to hand hygiene

The hands harbor greater bacterial diversity and are more temporally dynamic than other body sites [107]. More than 150 bacterial species have been recovered from human hands; these species primarily belong to the phyla Firmicutes, Actinobacteria, Proteobacteria, and Bacteroidetes [54, 107]. This increased diversity on human hands compared to other skin sites may be a result of the exposure of the hands to consistently varying external environments. Like other skin sites, there is a high degree of interpersonal variation in the hand microbiota; a minority of taxa (13%) are shared between the hands of any two individuals, and the two hands of a single person may share only a slightly larger fraction, though those communities appear somewhat stable [54] (but see [46], which suggests that resident communities are quite similar between dominant and non-dominant hands). Despite the evidence that certain bacterial species remain present on the hands over time, their relative abundances are variable [57]. Microbial communities on people’s hands are significantly affected by host factors, including sex, relatedness, living quarters, hand hygiene, and even pet ownership [54, 107, 108].

Hand hygiene is still regarded as the most important practice to prevent the transmission of microbes and minimize the spread of disease [109]. However, compliance with hand hygiene practices in healthcare settings is generally low, with mean baseline rates ranging from 5 to 89% [2]; typical rates may be no better than 40% [8, 109]. Current understanding of the effects of hand hygiene in healthcare settings largely stems from cultivation-based methods focusing on identification of pathogenic microbes. These clinical studies have historically been performed during periods of infectious outbreak in hospital settings with the assumption that bacteria on the skin are pathogenic contaminants [110]. Even with the growing use of high-throughput sequencing, there have been few cultivation-independent studies that have investigated the direct effects of hand hygiene or product use on the hand microbiota [46, 107, 111]. There is great potential to further our understanding of the human hand microbiota by utilizing an ecological perspective in healthcare settings, where hygiene practices are vital. Despite this current gap in knowledge, we are still able to draw preliminary conclusions about hand hygiene and its effect on the skin microbiota from cultivation-based studies and the few cultivation-independent studies that have looked at this relationship indirectly.

In cultivation-based studies, the length of direct patient contact is positively correlated with bacterial counts [109], and surface area and time of contact significantly affect the abundance of bacteria present on the hands of healthcare workers [112]. Older work has shown that soap and water handwashing is effective at removal of patient-acquired microbes [113], and more recent studies have shown alcohol-based handrubs to be as effective [109, 114] or even superior to soap and water [64]. There is also an interaction between skin health and the effect of hand hygiene that may be of concern: increased handwashing may increase the amount of microbes on the hands due to worsening skin health [46, 115]. Additionally, moisture level has a significant effect on cross-contamination rates [25–28]. However, these studies examined bacterial load on the hands and failed to address the *identities* of the species that were affected by hand hygiene practices—identity matters when most members of the microbiota are commensal or even potentially mutualistic.

Cultivation-independent studies show some similar trends: hand hygiene apparently reduced bacterial diversity on healthcare workers’ hands, though pathogen carriage on the hands was also observed [111]. Time since last handwashing was significantly correlated with changes in bacterial community composition but did not affect bacterial diversity [54]. This result could suggest that the microbiota present on the hands quickly reestablishes itself post-handwashing, or that few bacterial taxa are removed during the handwashing process [54].

In order for human-to-human microbial transmission to occur in a healthcare setting, the microbe must be capable of surviving for a period of time before it is transferred to another person or surface [116]. One study that looked at the transmission of *Klebsiella* spp. among healthcare workers in an intensive care unit found that only a slight contact with patients was needed to transfer the microbe to healthcare workers and that *Klebsiella* spp. could survive on dry hands for up to 150 min [117]. Another study found that the transmission potential of microbes to and from the hands and sterile fabrics was highly species-dependent, suggesting that hygienic practices may play a more vital role in transmission prevention of certain microbes over others [114].

From the available predominantly culture-dependent data, we can support that transmission via the hands is common and often related to microbial load and that variation in moisture levels affects transmission efficiency. However, since the majority of studies examined microbial load without taking taxonomic identities and ecological interactions into account, more studies are needed to quantify the role of interactions with the resident hand microbiota in the transmission of potentially pathogenic microorganisms.

## Redefining hygiene

Understanding the ecological dynamics within human-associated microbiota gives us the power to improve strategies for informed management of the crucial health-associated ecosystem services provided by these microbial communities. If the desired outcome of hygienic activities is to improve health and health is improved through optimal microbial maintenance and management within the host, then it would be beneficial to have hygienic guidelines that bear this in mind.

The evidence that microbes are essential for maintaining health supports the idea that hygienic practices aimed at the simple removal of microbes may not be the best approach. Rather, hygienic practices should aim to reduce *pathogenic* microorganisms and simultaneously increase and maintain the presence of *mutualistic* (or at least commensal) microorganisms essential for host protection. It is clear that microbial colonization of the skin is not deleterious, per se. Humans are covered in an imperceptible skim of microbial life at all times, with which we interact constantly. We posit that the conception of hygiene as a unilateral reduction or removal of microbial load has outlived its usefulness and that a definition of hygiene that is quantitative uses modern molecular biology tools and is focused on disease reduction is needed. As such, we explicitly define hygiene as “those actions and practices that reduce the spread or transmission of pathogenic microorganisms, and thus reduce the incidence of disease.”

It will, of course, be difficult to realize such a definition, given the great inertia of the modern hygiene/sterilization conflation [11–14], as well as the practical and ethical implications of human subjects research utilizing health outcomes as a dependent variable [118, 119]. Additionally, the inability to extricate causation from correlation in studies associating alteration of microbial community with particular disease manifestations hinders the ability to identify practices that reduce the spread specifically of pathogenic microorganisms [99–101, 104, 120–122].

These issues may be overcome through careful study design and application of new and emerging technologies. The power of explicitly defining hygiene in this manner is as a driver of future experimental design. A recent review by Kong et al. [44] outlines best practices for conducting skin microbiota studies, while a new study based on careful comparison of both cultivation-dependent and cultivation-independent methods from Zapka et al. [46] provides excellent recommendations specifically for hand microbiota studies as they relate to hygienic practices. To these recommendations, we would add that best practice in hygiene research should always consider health outcomes: the gold standard for demonstrating hygienic efficacy must be that a hygienic intervention reduces incidence of disease in the treatment group.

For example, a study examining differential hygienic efficacy of hand drying methods might install jet air dryers in a set nurses’ bathrooms and paper towel dispensers in another and then correlate drying method with the rate of nosocomial infections in patients seen by each nurse. Simultaneously, cultivation-independent detection methods could be used to track alterations to the microbiota of the nurses’ hands and the environment around them (nurses’ stations/bathrooms). This would allow the inference of a causal linkage between alterations in the microbiota, hand hygiene intervention, and patient health outcomes.

Understandably, this is a very high standard that will not always be possible to meet. There is still great value in studies that examine alterations in the microbiota without specifically testing health outcomes. However, researchers must avoid conflation of correlation and causation, as cautioned in [44]. Further elucidation of the disease dynamics and the natural history of common skin- or disease- associated microorganisms (e.g., [123]) will help greatly to facilitate a mechanistic understanding of the relationships between human microbial ecology and disease. Study designs that systematically track acquisition of new infections or diseases of interest in parallel to changes in the microbiota with application of hygiene interventions will, however, be the most valuable.

## Conclusions

Concepts of hygiene have evolved greatly over the last few centuries, influenced by cultural norms of cleanliness, empirical data, and the advent of the germ theory of disease. Through widespread acceptance of the germ theory, the common misconception that “all microbes are germs” has come to influence the modern conception of *hygiene*, such that it has become nearly synonymous with *sterilization*. The history of regulation of hygiene in healthcare-related settings generally reflects this usage. Modern microbial ecology using sensitive, cultivation-independent techniques provides a glimpse into the complexity of the microbial communities in, on, and around us, as well as a growing appreciation for the ecosystem services provided by these microbial communities.

Using such a definition alters the way we approach research on hygiene and suggests novel avenues. Studies of skin dysbioses [56, 61, 98] are beginning to demonstrate that consideration of species identity and ecological context is necessary to understand disease progression and devise effective treatments in some cases. Consideration of microbial ecological context as it relates to hygienic practice may improve understanding and treatment of many skin diseases, including atopic dermatitis, psoriasis, and acne. Already, methods similar to the gut microbe transplantation used to successfully combat *Clostridium difficile* infection are now under consideration for common skin diseases [53].

Very few studies of hand hygiene examine health outcomes, such as disease transmission or development of symptoms, as a dependent variable [19]. Nearly all studies of hand hygiene utilize bulk reduction in bacterial load as a proxy for reduced transmission of pathogenic organisms [2, 24]. However, due to the complex microbial ecology of the skin [55] and the potentially differential effects of such disturbances have on different microbial species [72], such a proxy is likely to not be broadly appropriate: it is necessary to know the identities and ecological roles of the organisms affected. New methods—including those that enable the assignment of functional groups to classes of microbes based on cultivation-independent, high-throughput DNA barcode surveys; quantification of the metabolically active portions of microbial communities and live/dead microbial determination methods; and high-throughput, whole-genome metagenomic sequencing, which enables the quantification and assignment of true functional potential—will help us to understand the ecological effects of hand hygiene practices. Explicit quantification of the effects of various hygienic practices on health metrics will allow us to understand the complex interplay between microbial community dynamics, hygienic practices, and health outcomes and, hopefully, provide meaningful data to support future recommendations and regulations for hygiene practices.

**Box 1:** Glossary

*Biogeography*—the discipline studying the distribution of species and ecosystems in space and across evolutionarily meaningful timescales.
*Community ecology*—the discipline studying the organization and function of ecological communities (those organisms actually or potentially interacting, bounded by either geographic or conceptual limits).
*Contamination*—incidental presence of microbes; not long-term residents of the microbial ecosystem in question.
*Cultivation-dependent detection*—microbiological techniques that rely on the cultivation of microbes for enumeration and identification; less than 1% of microbes are estimated to have been cultivated in the lab [41, 42], leaving a vast majority of microbial diversity underexplored (Fig. 1d).
*Cultivation-independent detection*—techniques for the elucidation of microbial communities that do not rely on cultivation of microorganisms; these generally rely on high-throughput, next-generation sequencing technologies (e.g., Illumina, 454 pyrosequencing) that allow for the direct sequencing of DNA from the environment; common techniques include *metabarcoding*, in which a conserved “barcode” region of the genome is amplified and sequenced from environmental samples, giving information about which taxa are present and their relative abundances, and *metagenomics*, in which all available microbial DNA is sequenced, giving information about presence and relative abundances of metabolic pathways as well as identities of microbes (Fig. 1e).
*Dispersal*—the distribution of propagules across space.
*Dysbiosis*—an alteration of the microbiota from the norm, generally associated with disease states; this may be through shifts in dominance or the addition/removal of key taxa.
*Environmental filtering*—the process by which potential colonists are selected based on purely ecological factors.
*Ecological niche—*a broad term encompassing multiple definitions used to describe to an organism’s activity or behavior in response to a given set of biotic and abiotic environmental conditions or resources. Organisms occupy niches by carrying out specific functions, often through competitive or mutualistic interactions. *Niche space* refers to the set of all possible niches, occupied or unoccupied, in a given habitat.
*Hygiene*—those actions and practices that reduce the spread or transmission of pathogenic microorganisms, and thus reduce the incidence of disease.
*Hygiene hypothesis*—the idea that a lack of early childhood exposure to microorganisms increases susceptibility to allergic diseases by suppressing the natural development of the immune system.
*Invasion ecology*—the discipline studying the alterations to ecosystems resulting from introduction and establishment of taxa originating outside of said ecosystem, and the factors allowing some taxa to invade successfully.
*Microbial ecology*—the discipline studying the interrelations between microorganisms, including but not limited to community interactions and interactions with the environment.
*Microbial load*—the absolute abundance of microbes; commonly estimated using cultivation-dependent techniques through quantitative counts of *colony-forming units* (CFUs).
*Microbiota*—or *microbiome*, the ecological community of microorganisms (bacteria, archaea, viruses, fungi, mites, etc.) that share our body space; may be subdivided into cohesive groups, such as the skin microbiota, or the gut microbiota.
*Nosocomial*—of or relating to hospitals.
*Priority effects*—the particular influence that early arriving members of a community have on later arriving members.
*Protective mutualism*—a mutualism in which protection from pathogenic organisms is the result of occupation of niche space within the host habitat, excluding colonization by harmful microbes; often conflated with commensalism (see Fig. 2c).
*Sterilization*—the removal of all microbes from a surface or object.
*Transmission*—dispersal and establishment of microbes between hosts.

## Abbreviations

CDD: Cultivation-dependent detection
CFU: Colony-forming unit
CID: Cultivation-independent detection
